# Baicalin alleviates benign prostate hyperplasia through androgen-dependent apoptosis

**DOI:** 10.18632/aging.102731

**Published:** 2020-02-04

**Authors:** Bo-Ram Jin, Hyo-Jin An

**Affiliations:** 1 Department of Pharmacology, College of Korean Medicine, Sangji University, Wonju-si 26339, Gangwon-do, Republic of Korea

**Keywords:** benign prostatic hyperplasia, androgen, baicalin, proliferation, apoptosis

## Abstract

BPH is a disease prevalent among elderly men that is characterized by abnormal proliferation of prostatic epithelial and stromal tissues. No effective treatment exists for BPH owing to lack of a clear understanding of its molecular etiology. Although several studies have reported therapeutic effects of baicalin against numerous diseases, including prostate cancer, its beneficial effects on BPH have not yet been explored. The present study investigated the therapeutic effects of baicalin on the development of BPH and its mechanism of action. We established a testosterone-treated BPH animal model and DHT-stimulated prostate cell lines, including RWPE-1 and WPMY-1. Administration of baicalin ameliorated the pathological prostate enlargement, suppressed the production of DHT, and inhibited the activity of 5α- reductase Type II in the animal model. BC exerted these effects via its anti-proliferative effects by restoring the Bax/Bcl-2 ratio, activating caspase-3 and caspase-8, and inducing the phosphorylation of AMPK. *In vitro* studies using DHT-stimulated prostate cells demonstrated an up-regulation of BPH-related and proliferation markers, whereas baicalin clearly reduced the overexpression of AR, PSA, PCNA, and Bcl-2. These results suggested that baicalin could suppress androgen-dependent development of BPH both *in vivo* and *in vitro* by inducing apoptosis.

## INTRODUCTION

Benign prostate hyperplasia (BPH) and prostate cancer (PCa) are age-related chronic diseases that initiate at an early age and progress slowly [1]. Several clinical studies have suggested a link between BPH and PCa owing to a number of similarities between the two. It has been speculated that two diseases have similar clinical presentation and pathogenic mechanisms. Especially, both are characterized by rapid proliferation of prostatic cells. In contrast, other studies have reported no association between these. Despite the existence of a lot of controversy between the two diseases, common pathophysiological risk factors include chronic inflammation, metabolic disturbances, and hormonal association [2, 3]. Moreover, epidemiological studies have suggested that since the incidence and prevalence of two diseases increase with age, their pathoprogression is hormone dependent and involved in chronic prostatic inflammation [4].

BPH, defined as a nonmalignant expansion of the prostate gland, is one of the most prevalent diseases in elderly men. The incidence of BPH is reported to be 20% in men in the age group of 40 to 60 years and 90% in those above 80 years of age [5]. The enlarged prostate gland causes bladder outlet obstruction and lower urinary tract symptoms (LUTS), which seriously damage the quality of life. LUTS suggestive of BPH include urinary frequency, urgency, nocturnal enuresis and double voiding [6]. Based on the histological diagnosis, BPH has been characterized by abnormal proliferation of epithelial and stromal cells of the prostate gland, with the extent of proliferation of epithelial and stromal cells being 9- and 37-fold, respectively, as compared to the proliferation capacity of the normal prostate cells [7].

Alteration in the levels of androgens is postulated to be the primary cause of development and progression of prostate diseases, including BPH. Despite the androgen-dependent prostate cell proliferation, a clear understanding of its downstream mechanisms is still lacking. Nevertheless, blockade of androgen signaling forms the basis of pharmacological treatment of BPH [8]. Pharmaceutical drugs, including 5α-reductase inhibitors and α-adrenergic blockers have proved effective in treating BPH; however, these therapies are not completely beneficial and exhibit certain side effects, including insomnia, erectile dysfunction, and subsequent progression to prostate cancer. Hence, there is a need to develop substitutional drugs that could exert long-term therapeutic effects and reduce undesired problems in clinical use. Therefore, an increasing number of studies are being conducted to develop new drugs from natural sources, with potential therapeutic effects and fewer side effects.

Baicalin (BC) (7-D-glucuronic acid-5,6-dihydroxyflavone) is a flavonoid extracted from the dried roots of *Scutellaria baicalensis* Georgi which has been clinically utilized as the traditional medicines and the nutritional supplements [9]. BC is known for its several pharmacological actions, including anti-inflammatory and anti-allergic properties [10, 11]. Studies using several cell lines and animal models have reported BC or herbal extracts rich in BC to possess anti-proliferative or anti-tumor effects [12–14]. Interestingly, some studies have reported that the therapeutic efficacy of BC against various diseases may be closely associated with apoptosis signaling pathway. A previous research also suggested that BC could inhibit growth and viability of prostatic cancer cells by inducing apoptosis [15]. Moreover, our previous study found that *S. baicalensis* Georgi could inhibit the development of BPH by targeting inflammation- and apoptosis-related markers [16]. However, the therapeutic effects on BPH and underlying molecular mechanism(s) of BC is still not known.

The present study was designed to explore the effects of BC on the development of BPH using *in vivo* and *in vitro* models. Testosterone propionate (TP)-treated BPH animal model greatly emulates the histological characteristics of human BPH and is used to assess the efficacy of various drug candidates. Using the BPH-induced rat model, we demonstrated the anti-BPH effects of BC and investigated the involved molecular mechanism(s). Moreover, we established dihydrotestosterone (DHT)-stimulated prostate cell lines, including RWPE-1 and WPMY-1, to study the action of androgens in normal prostate cell lines and evaluate the inhibitory effect of BC on BPH-induced cell lines.

## RESULTS

### BC prevented enlargement of prostate and morphological changes in TP-treated BPH rat model

**Figure 1:**
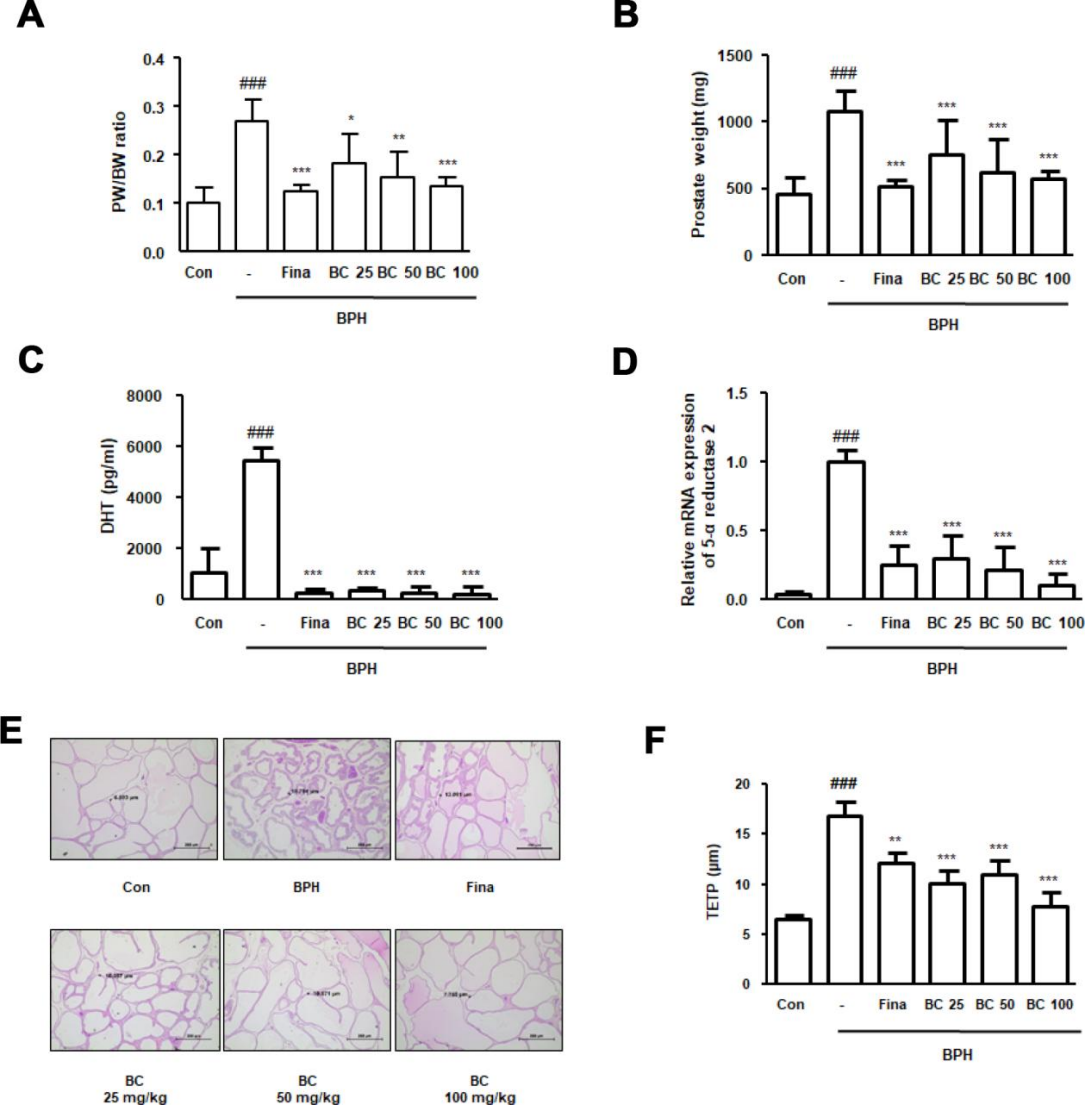
**Effect of BC on prostatic enlargement by inhibition of 5α-reductase Type II in TP-treated BPH rat model.** Animals were injected with TP for 4 weeks with or without Fina and various concentration of BC. (**A**) Prostate weight to body weight (PW/BW) ratio and (**B**) prostate weight in each group were assessed. PW/BW ratio was calculated as (average prostate weight of the experimental group/ average body weight of the experimental group) × 1000. (**C**) DHT serum concentrations were analyzed using ELISA kit. (**D**) 5α-reductase Type II mRNA expression was showed by qRT-PCR analysis in prostatic tissues. (**E**) H&E staining analysis was performed using prostatic tissue sections. Original magnification 100x. (**F**) Based on H&E staining, TETP was measured and represented. All data are shown as the average value of each experimental group and are mean ± SD (n = 8). P value ^###^ = P < 0.001 versus Con group; * = P < 0.05, ** = P <0.01, *** = P < 0.001 versus the BPH group.

Numerous studies have used TP-treated rats as a model to study BPH owing to its characteristic resemblance to the actual disease [17, 18]. To evaluate the therapeutic effects of BC, rats were administered TP for 4 weeks, with or without Fina and BC. As shown in Figure 1A and 1B, rats in the BPH group had a significantly increased prostate weight and prostate weight to body weight ratio compared to rats in the control group. In contrast to the BPH group, treatment with Fina, BC 25, BC 50, and BC 100 significantly reduced the enlarged prostate volume by 43.76%, 21.27%, 27.23%, and 34.77%, respectively. To determine the effect of hormonal imbalance on the suppression of prostate enlargement by BC, we assessed the serum DHT levels in each experimental group. As compared to the control group, abnormal DHT levels were detected by ELISA in the BPH group. Data revealed that administration of Fina, BC 25, BC 50, and BC 100 significantly alleviated the overproduction of DHT induced by TP treatment (Figure 1C). In males, about 70% of DHT is derived from testosterone by 5α–reductase in the prostate, testes, and adrenal glands. Abnormal levels of DHT may result in development of BPH, as well as PCa. Results from 5α-reductase type 2 mRNA expression study were consistent with the data obtained from the study of DHT levels. As shown in Figure 1D, administration of Fina, BC 25, BC 50, and BC 100 clearly inhibited the mRNA expression of 5α-reductases type 2 in comparison to the BPH group. Next, we conducted histological analysis and measured the thickness of the epithelium from prostate tissue (TETP) using H&E staining. As can be seen from Figure 1E, the BPH group revealed archetypal hyperplastic patterns, such as a thickened muscle layer, varifold layer of epithelium, and reduced inside space of a tubular structure, whereas administration of Fina, BC 25, BC 50, and BC 100 inhibited the prostate hypertrophy. TETP following induction of BPH was significantly increased up to 2.55 times as compared to its thickness in the control group, whereas administration of Fina, BC 25, BC 50, and BC 100 partially reduced the thickness in comparison to that observed in the BPH group by 27.95%, 40.10%, 34.62%, and 53.81%, respectively (Figure 1F).

### BC attenuated TP-treated BPH via inhibition of inflammatory proteins

**Figure 2:**
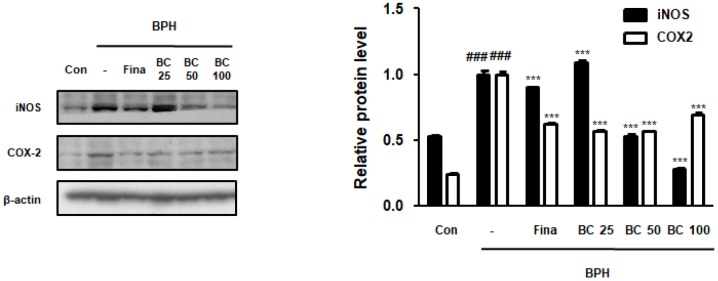
**Effect of BC on inflammatory makers in TP-treated BPH rats model.** Prostatic tissue lysates were immunoblotted with iNOS and COX-2 to investigate effect of BC on compensatory cellular proliferation in TP-treated BPH rat model. β-actin served as an internal control. Fold changes in densitometric analysis was normalized to β-actin and are represented as mean ± SD, which are acquired Image J. P value ^###^ = P < 0.001 versus Con group; *** = P < 0.001 versus the BPH group.

Previous studies have demonstrated the role of inflammation in BPH, suggesting that a number of inflammatory factors may be implicated in BPH and associated symptoms. For example, overexpression of iNOS has been reported in prostate tissues with BPH. NO enhances the activity of COX-2, which is abundantly present in the epithelial and stromal cells of the human prostate gland and is responsible for inducing inflammation [19]. As seen from Figure 2, iNOS levels were higher in the BPH group than its levels in the control group, whereas administration of Fina, BC 50, and BC 100 significantly attenuated the overexpression of iNOS protein. In addition, COX2 expression in the total prostatic protein increased in the BPH group, whereas Fina, BC 25, BC 50, and BC 100 clearly alleviated its expression.

### BC inhibited prostate cellular proliferation in TP-treated BPH rat model

**Figure 3:**
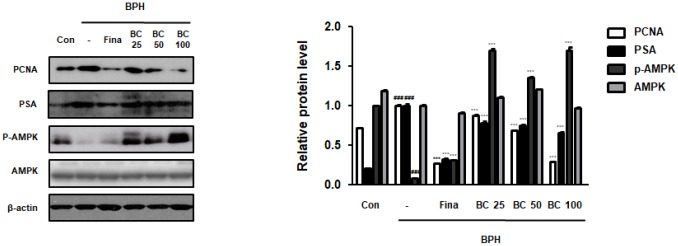
**Effect of BC on the cell proliferation in TP-treated BPH rats model.** Immunoblot results showed the level of PCNA, PSA, p-AMPK, AMPK in prostatic tissues. Densitometric protein levels of PCNA, PSA, p-AMPK, AMPK are represented as mean ± SD and plots of each protein were showed. P value ^###^ = P < 0.001 versus Con group; *** = P < 0.001 versus the BPH group.

PCNA is a well-known cell proliferation marker that functions as a cofactor in DNA replication during the S phase of the cell cycle [18]. PSA levels, used in the clinical judgment of PCa, are also being used to provide accurate evidence on prostate volume and risk of its progression in BPH [20]. We investigated whether BC could inhibit the expression of PCNA and PSA proteins in TP-treated BPH rats. In contrast to the control group, BPH group showed an overexpression of PCNA and PSA proteins, whereas BC exerted a dose-dependent inhibitory effect. AMPK activation induces intrinsic apoptosis pathway and blocks mitosis, indicating it to be regulating the cell cycle beyond its metabolic activity [21]. Here, we analyzed the effect of BC on AMPK activation. The BPH group showed significantly reduced phosphorylation of AMPK in comparison to the control group; however, it did not affect the total AMPK expression. In contrast, BC 25, BC 50, and BC 100 clearly restored AMPK phosphorylation, suggesting inhibitory effects of BC on BPH via AMPK-dependent pathways (Figure 3).

### BC suppressed TP-treated BPH via normalization of abnormal balance between proliferation and apoptosis

**Figure 4:**
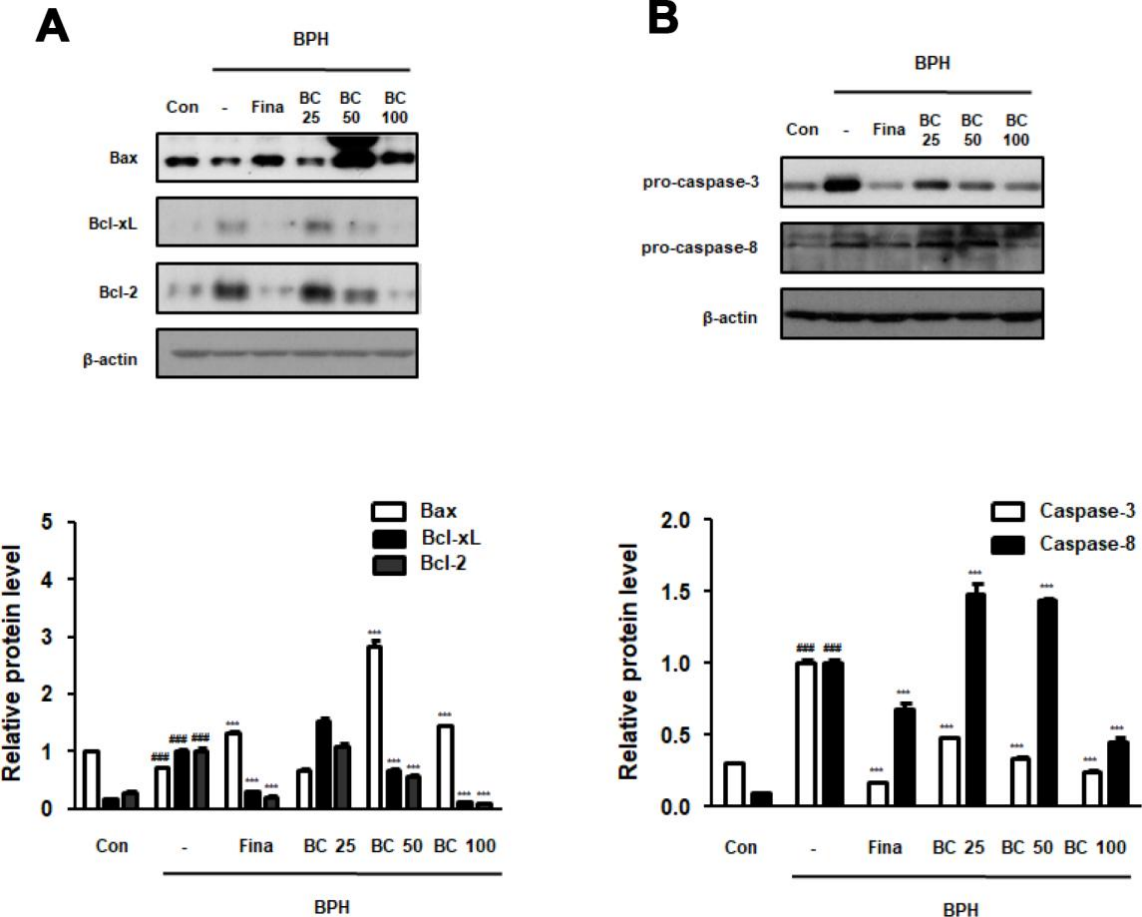
**Effect of BC on apoptosis regulatory proteins in TP-treated BPH rats model.** (**A**) The protein levels of Bcl-2 gene family and (**B**) pro-caspase-3 and pro-caspase-8 were determined via western blot using specific antibodies in prostatic tissue lysates. β-actin served as an internal control. Densitometric analysis on each protein was performed and relative protein levels were represented as mean ± SD. P value ^###^ = P < 0.001 versus Con group; *** = P < 0.001 versus the BPH group.

Disruption of the balance between proliferation and apoptosis in prostate cells is associated with the excessive growth and development of prostate cells. Apoptotic rate has been reported to be higher in normal tissues than in BPH tissues, whereas a significantly higher proliferation rate is observed in the hyperplastic prostate [22]. Here, to demonstrate the underlying molecular mechanisms of prostatic hyperplasia, we evaluated the expression of crucial proliferation-related markers. We found that the balance of Bax to Bcl-2 was significantly impaired by BPH induction, whereas Fina, BC 50, and BC 100 administration clearly restored this balance. Correspondingly, Bcl-xL, one of the members of the Bcl-2 family, showed an increased expression in the BPH group as compared to its expression in the control group, whereas administration of Fina, BC 50, and BC 100 attenuated the effect of TP treatment. Mitochondrial apoptosis pathway proteins, including those belonging to Bid and Bcl-2 families, could be activated through activation of caspase-8 and caspase-3 (receptor pathway) [23]. We then tested whether anti-BPH effect of BC via restoration of Bax/Bcl-2 balance was linked to an association between the receptor and mitochondrial pathway. In the BPH group, the expression of procaspase-8 was significantly increased as compared to its expression in the control group, whereas administration of Fina and BC 100 clearly reduced the protein expression. In addition, Fina, BC 25, BC 50 and BC 100 reduced the inactive form of procaspase-3, which is directly affected by caspase-8 as a downstream effector (Figure 4).

### BC inhibited BPH-related protein and mRNA levels in DHT-treated RWPE-1 cells

**Figure 5:**
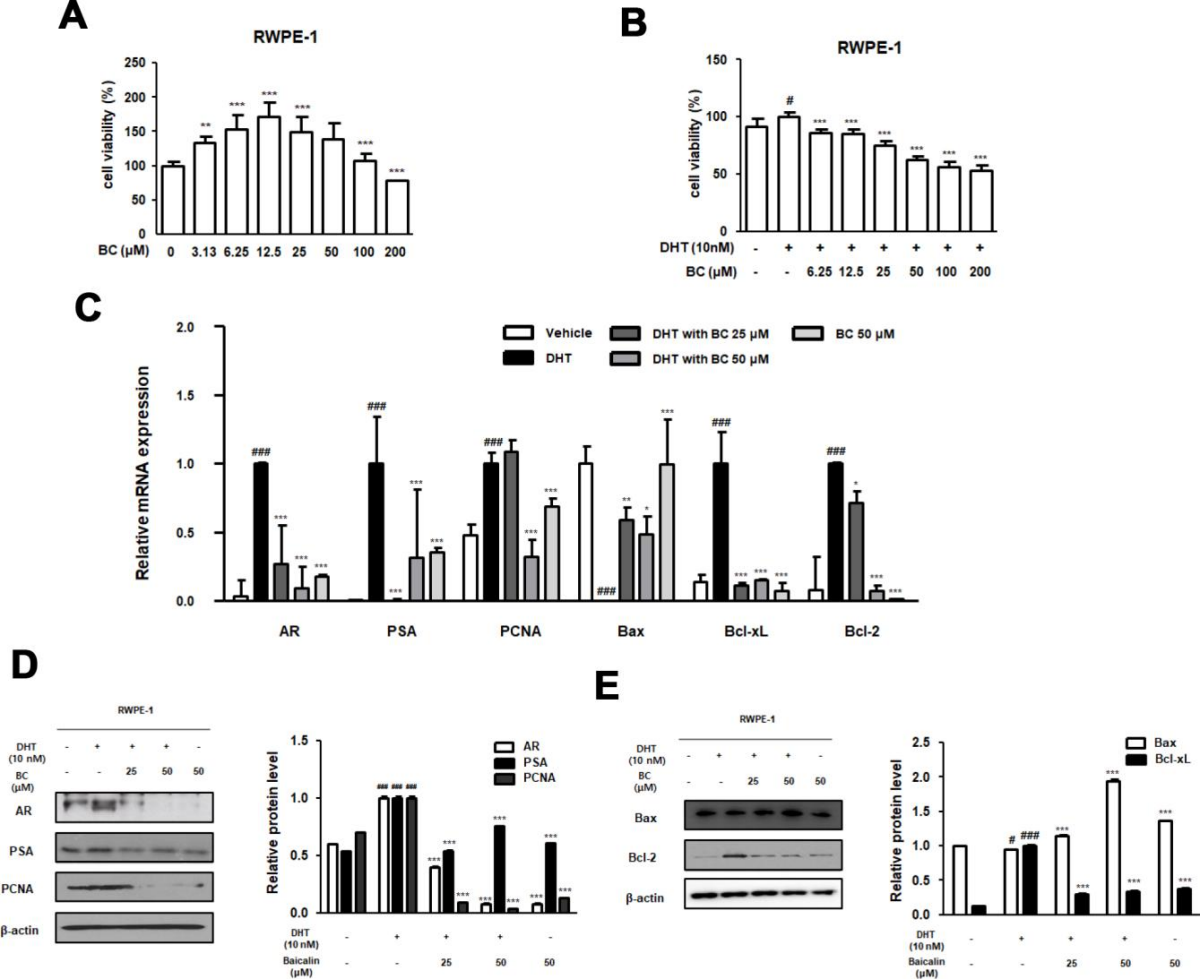
**Effect of BC on the BPH-related and apoptosis related protein and mRNA expression in RWPE-1 cells.** (**A**) Effect of BC on the cell viability in RWPE-1 cells. RWPE-1 cells were treated with 3.13 to 200 μM of BC for 24 h. (**B**) Inhibitory effect of BC on cell proliferation in DHT-stimulated RWPE-1 cells. RWPE-1 cells were treated with 10 nM DHT, with or without 6.25–200 μM of BC for 24h. (**C**–**E**) RWPE-1 were stimulated with 10 nM DHT from 3days to 5days, with or without BC (25, 50 μM). (**C**) The mRNA level of AR, PSA, PCNA, Bax, Bcl-xL and Bcl-2 were quantificated using RT-PCR. RWPE-1 cell lysates were immunoblotted with (**D**) AR, PSA, PCNA (**E**) Bax, Bcl-2 primary antibodies. Protein levels which are normalized by internal control β-actin are represented as relative protein levels. P value ### = P < 0.001 versus vehicle group; * = P < 0.05, ** = P <0.01, *** = P < 0.001 versus the DHT-stimulated group.

To demonstrate the inhibitory effect of BC on cellular proliferation, we established an in vitro model of BPH by treating normal prostate epithelial RWPE-1 cells with DHT. We first confirmed the effective dose of BC that did not affect cell viability. As shown in Figure 5A, MTS assay demonstrated that treatment of cells with BC (3.13–200 μM) for 24 h resulted in no toxicity. Using CCK-8 assay, we tested the cell proliferation effect of DHT on RWPE-1 cells and evaluated inhibitory effect of BC. In the DHT 10 nM treated cells, the rate of cell proliferation was significantly increased as compared to its rate in the untreated cells. However, BC (6.25-200 μM) treatment significantly inhibited the rate of cell proliferation with DHT treatment in RWPE-1 cells. Next, we used qRT-PCR and western blot analysis to investigate the anti-BPH effects of 25 and 50 μM BC on DHT-stimulated RWPE-1 cells via studying the apoptosis signaling pathway. In RWPE-1 cells, DHT stimulation caused an overexpression of AR, PSA, PCNA, Bcl-xL and Bcl-2 mRNAs, and down-regulation of Bax mRNA. However, treatment with 25 and 50 μM BC alleviated DHT-stimulated abnormal expression of BPH and proliferative markers at the mRNA level (Figure 5C). Consistent with these results, the protein expression of AR, PSA, PCNA and Bcl-2 significantly increased after DHT treatment. In contrast, treatment with 25 and 50 μM BC significantly inhibited the overexpression of these proteins and restored the expression of Bax protein, which was impaired by DHT stimulation (Figure 5D and 5E).

### BC suppressed BPH-related protein and mRNA levels in DHT-treated WPMY-1 cells

**Figure 6:**
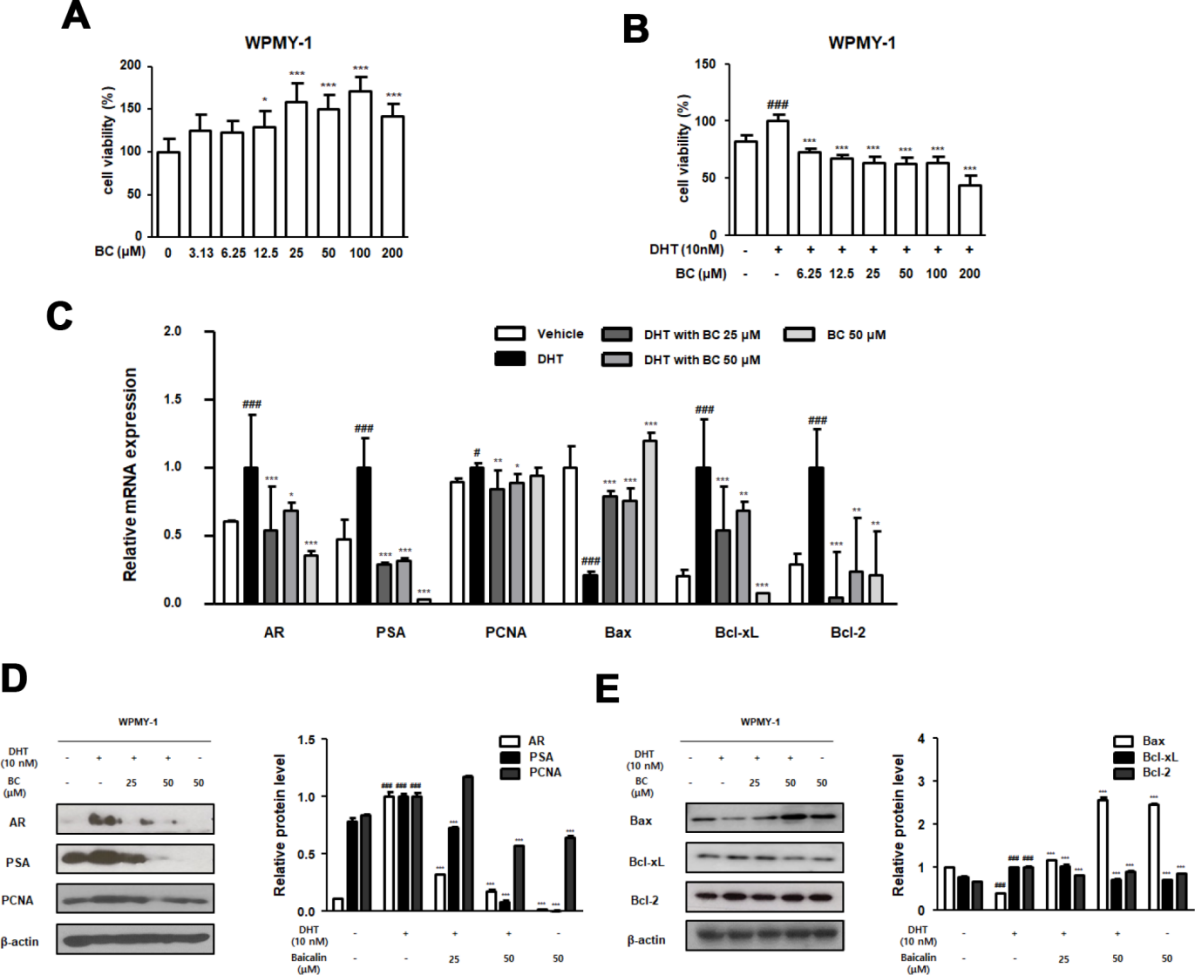
**Effect of BC on the BPH-related and apoptosis related protein and mRNA expression in WPMY-1 cells.** (**A**) Effect of BC on the cell viability in WPMY-1 cells. WPMY-1 cells were treated with 3.13 to 200 μM of BC for 24 h. (**B**) Inhibitory effect of BC on cell proliferation in DHT-stimulated WPMY-1 cells. The cells were treated with 10 nM DHT, with or without 6.25–200 μM of BC for 24h. (**C**–**E**) WPMY-1 cells were stimulated with 10 nM DHT for 24h, with or without BC (25, 50 μM). (**B**) The mRNA level of AR, PSA, PCNA, Bax, Bcl-xL and Bcl-2 were quantificated using RT-PCR in DHT-stimulated WPMY-1 cells. Protein levels of (**C**) AR, PSA, PCNA (**D**) Bax, Bcl-xL and Bcl-2 are determined using western blotting in DHT-stimulated WPMY-1 cells. β-actin are used for internal control. Results are represented as mean ± SD. P value ### = P < 0.001 versus vehicle group; * = P < 0.05, ** = P <0.01, *** = P < 0.001 versus the DHT-stimulated group.

Figure 5 shows that BC inhibited the mRNA and protein levels of irregular BPH-related and anti-apoptotic markers in DHT-stimulated prostate epithelial RWPE-1 cells. We next identified the inhibitory effect of BC on DHT-stimulated prostate stromal cell WPMY-1. As evident from Figure 6A, treatment with BC (3.13–200 μM) had no effect on WPMY-1 cell toxicity. Next, we examined whether BC suppresses DHT-stimulated cellular proliferation in WPMY-1 cells. We measured the extent of cell proliferation utilizing the CCK-8 assay and confirmed the anti-proliferation effect of 6.25–200 μM BC in DHT-treated WPMY-1 cells (Figure 6B). Treatment of WPMY-1 cells with DHT up-regulated the expression of AR, PSA, PCNA, Bcl-xL and Bcl-2 and down-regulated the expression of Bax mRNAs (Figure 6C). A similar trend was observed in the expression of these proteins (Figure 6D and 6E). As expected, treatment with 25 and 50 μM BC reduced the mRNA levels and protein expression of AR, PSA, PCNA, Bcl-xL and Bcl-2, and restored that of Bax.

## DISCUSSION

Several studies have explored factors influencing BPH, such as hormone imbalance, metabolic syndrome, age, and chronic inflammation; however, not much is known about its etiology. Among these factors, increase in the levels of androgens has been postulated to be linked to BPH pathogenesis. Androgens, including testosterone, play a central role in the development and differentiation of the prostate gland. AR is a nuclear receptor that functions as a transcription factor. It binds to several ligands, such as testosterone and DHT, and controls the expression of various target genes including PSA, which, in turn, regulates the growth and survival of prostate cells [24, 25]. Aberrant activation of AR by androgens causes alterations in proliferation, apoptosis, and angiogenic events, eventually causing BPH or PCa. The significant role played by androgens in the pathogenesis of BPH and PCa has led androgen deprivation therapy to be the mainstay of the treatment [26]. For example, 5α-reductase inhibitors, such as finasteride and dutasteride, are used to treat symptomatic nodular hyperplasia and enlarged prostatic volume. However, their use is restricted owing to the associated side effects, such as erectile dysfunction and loss of libido. These side effects appear in the early stage and sometimes persist even after ending the therapy [27]. Therefore, the major challenge faced by the pharmaceutical industry worldwide is to develop alternatives to minimize the undesired effects [28]. In this regard, phytochemicals, such as β-sitosterol and cernilton, and those based on saw palmetto and *Pygeum africanum*, have emerged as the new treatment choices. [29, 30]. Although therapeutic potential of herbal medicines has been supported by an increased number of literature and clinical trials, much uncertainty still exists about their pharmacological functions and underlying molecular mechanism(s).

Baicalin is a major constituent of *S. baicalensis* Georgi that has been widely used as a traditional Chinese medicine for treating high fever, external bleeding, and inflammation [31]. Findings of the current study demonstrated that *S. baicalensis* exerted suppressive effect on cell proliferation via repression of 5-lipoxygenase activity, supporting the findings by previous studies and suggesting that this plant could serve as a potential source of anti-cancer drugs [32]. Further investigations have revealed that the extract of this plant has cytotoxic effects on several cancer cells, including prostate cancer cells [33, 34]. Another study reports the anti-tumor effects of BC on human prostate cancer cells via induction of apoptosis [15].

Numerous studies point toward an association between BPH and PCa. BPH and PCa are considered as leading causes of urinary tract disorders in men; both are characterized by rapid proliferation of prostatic cells. It has been speculated that two diseases have similar clinical presentation and pathogenic mechanisms [35]. Based on this hypothesis, in previous studies, we investigated the therapeutic effects of *S. baicalensis* Georgi on BPH via inhibiting inflammation and proliferation-related markers in TP-treated BPH rat model. In the present study, we confirmed that BC inhibited prostate enlargement and overproduction of DHT in TP-treated BPH rats. Because the level of DHT correlated with the expression of 5α-reductase type 2, BC could be regarded as a potential therapeutic agent that exerts its effects via controlling androgen and 5α-reductase type 2 levels. Moreover, therapeutic effects of BC on abnormal proliferation were verified by its ability to reduce the thickness of prostatic muscle layer and prostatic hypertrophy (Figure 1). We also demonstrated the inhibitory effects of BC on the expression of iNOS and COX-2 proteins in TP-treated BPH rats (Figure 2). These observations are supported by previous studies that report *S. baicalensis*-mediated inhibition of cancer cell proliferation through reduction of COX-2 expression [34]. Furthermore, several studies have demonstrated chronic inflammation and development of prostatic disease to be linked, demonstrating overexpression of COX-2 protein and cell proliferation-related makers in prostatic epithelium of BPH and PCa [36]. Similarly, increased expression of iNOS that generates NO and enhances COX activity was detected in BPH and low- and high-grade prostatic intraepithelial neoplasia samples [37].

Anti-proliferative effects of BC against a wide spectrum of diseases have been suggested to be mediated through augmentation of AMPK. AMPK not only senses the changes in cellular energy levels, but also responds to reactive oxygen species and activates processes such as apoptosis and autophagy [38]. A recent study demonstrated AMPK-mediated inhibition of cancer cell proliferation by BC [39]. As shown in Figure 3, we observed an altered AMPK phosphorylation in the BPH group as compared to the control group. However, administration of BC restored the levels of AMPK phosphorylation. Moreover, a significant increase in the proliferation marker, PCNA, which is associated with DNA damage and repair, was observed in the tissues of patients with PCa and BPH [40]. PSA is another protein that is overexpressed during PCa development and along with PCNA is one of the most extensively used biomarkers for PCa and BPH [41]. In line with the mentioned studies, the present study found PCNA and PSA proteins to be overexpressed in the BPH group, whereas these effects were abolished by treatment with BC in a dose-dependent manner.

The intrinsic apoptosis pathway is associated with regulation of Bcl-2 family, which comprises anti-apoptotic proteins such as Bcl-2 and Bcl-xL, and pro-apoptosis effectors such as Bax [42]. We found that administration of BC 50 and 100 greatly restored the expression of Bcl-2, Bcl-xL and Bax to the control levels (Figure 4A). Bcl-2 regulates caspase-3 cascade, finally preventing apoptosis by functioning at a point downstream from release of mitochondrial cytochrome c [43]. As shown in Figure 4B, restored procaspase-3 expression in the group administered BC could be attributed to regulation of a series of intrinsic mitochondrial-dependent apoptosis cascade. Meanwhile, the restored caspase-8 levels in the group treated with BC indicate that administration of BC might exert its effects through the extrinsic apoptosis pathway that is executed via death receptor to caspase-8, finally activating caspase-3 [44].

In the present study, we investigated the effect of BC on DHT-stimulated RWPE-1 and WPMY-1 prostate cell lines and its mechanism of action in detail. The non-tumorigenic human prostatic epithelial RWPE-1 cells exhibit growth and differentiation that are characteristics of the normal prostatic epithelium [45]. The WPMY-1 cell line, derived from human prostatic stromal myofibroblast cells, has been widely used as a model for studying interaction between stromal and epithelial cells in prostate diseases [46]. These cells were treated with DHT to induce BPH *in vitro* and served as a good model owing to androgen responsiveness and expression of markers of BPH. Based on our results, we conclude that DHT-stimulated RWPE-1 and WPMY-1 cells induced overexpression of AR protein and mRNA. The role of AR is to enhance cell proliferation and survival of glandular epithelium of the prostate, such that its overexpression contributes to progression of prostate diseases. Although androgens directly act on the epithelial cells via epithelial AR to evoke differentiation, several studies have reported these to function indirectly via stromal AR [47]. Stromal AR is known to be associated with development and growth of prostate both during fetal life and prostate pathoprogression [48]. Results from several studies are consistent with the proliferative function of AR in DHT-stimulated prostate cells. The protein and mRNA levels of PSA and PCNA were also significantly increased and were inhibited by BC treatment in DHT-stimulated RWPE-1 and WPMY-1 cells. Furthermore, aberration in the levels of Bcl-family genes in DHT-stimulated RWPE-1 and WPMY-1 cells was restored with BC treatment (Figures 5 and 6), supporting that survival effects of AR connote expression of anti-apoptotic protein Bcl-2 [49].

## CONCLUSION

BC effectively ameliorated TP-treated BPH in rat models and DHT-treated RWPE-1 and WPMY-1 cells by regulating the androgen-dependent proliferation. BC exerted these effects through activation of intrinsic apoptosis pathway, leading to inhibition of 5α-reductase type 2 activity and DHT production. Moreover, inhibitory effects of BC on DHT-stimulated prostatic cells were exerted via interaction between AR and anti-apoptotic proteins. To the best of our knowledge, present study is the first of its kind to provide evidence of therapeutic effects of BC on androgen-proliferation signaling using TP-treated BPH rat model and DHT-treated RWPE-1 and WPMY-1 cells.

## MATERIALS AND METHODS

### Animals

Male Sprague-Dawley rats weighing between 180 and 220 g (6 weeks of age) were obtained from Dae Han Bio Link Co., Daejeon, Korea. All animal studies were performed according to the guidelines for the care and use of laboratory animals established by the National Institutes of Health. The Institutional Animal Care and Use Committee (IACUC) of the Sangji University certified and approved all animal experimental protocols (no. 2014-23).

### Induction of BPH and drug administration

Rats were castrated by intraperitoneal administration of Zoletile 50. Rats in the control group (Con group) were cut open and sewed up without castration. BPH was induced in rats by subcutaneous injections (s.c.) of TP (Wako Pure Chemicals, Tokyo, Japan) for 4 weeks after castration. TP-treated rats were divided into five groups and orally injected with water (BPH group), finasteride (Merck and Co., Inc., NJ, USA) 5 mg/kg (Fina group), baicalin (CAS Number: 21967-41-9, Sigma-Aldrich, MO, USA) 25, 50, and 100 mg/kg (BC 25, 50, 100) for 4 weeks except weekends. On day 28 after the first TP injection, all rats were sacrificed by anesthesis with Zoletil® 50 (intraperitoneal, 20 mg/kg; Virbac, France), and the prostatic tissues were excised, weighed, and stored at – 80°C.

### Serum concentrations for DHT analysis

Blood samples were collected from all experimental animals, and serum was separated using a Vacutainer tube (Becton Dickinson, NJ, USA). The serum DHT levels were determined using a commercial enzyme-linked immunosorbent assay (ELISA) kit (CUSABIO; TX, USA). The assay was performed according to the manufacturer’s instructions.

**Table 1:** Primer sequences.

**Gene name**	**Forward primers (5′-3′)**	**Reverse primers (5′-3′)**
**rats 5α-reductase 2**	GGC AGC TAC CAA CTG TGA CC	CTC CCG ACG ACA CAC TCT CT
**rats GAPDH**	TGA TTC TAC CCA CGG CAA GT	AGC ATC ACC CCA TTT GAT GT
**human AR**	GAGCCAGGTGTAGTGTGTGC	TCGTCCACGTGTAAGTTGCG
**human PSA**	ATAGGATTGCCCAGGCAGAA	CTAAGGGTAAAAGCAGGGAGAGAGT
**human PCNA**	TTAAACGGTTGCAGGCGTAG	AGGAAAGTCTAGCTGGTTTCGG
**human Bax**	ATGCGTCCACCAAGAAGCTG	AACATGTCAGCTGCCACTCG
**human Bcl-xL**	ATCCACTCTACCCTCCCACC	GGGAGTGAGGACTCTAGCCA
**human Bcl-2**	CCATGTTGTTGGCCGGATCA	TCTTCTTCAGGCCAGGGAGG
**human β-actin**	GGCCAGGTCATCACCATTGG	CTTTGCGGATGTCCACGTCA

### Histological analysis

The prostate tissues in each group were fixed with 4% formalin and embedded in paraffin. The tissues were then cut into 4-mm sections. The sections were stained with hematoxylin and eosin (H&E) for histological examination. Images were acquired using a Leica microscope (Leica DFC295; Wetzlar, Germany).

### Quantitative real-time polymerase chain reaction analysis

Total RNA was isolated using the Easy-Blue® reagent (iNtRON Biotechnology, Inc., Gyeonggi-do, Korea) and transcribed into cDNA according to the manufacturer’s instructions. Quantitative real-time polymerase chain reaction (qRT-PCR) was performed as described [50].

### Western blot analysis

Antibodies against inducible nitric oxide (NO) synthase (iNOS; sc-650), cyclooxygenase-2 (COX-2; sc-1745), Bax (sc-7480), Bcl-xL (sc-8392), Caspase-3 (sc-7272), Caspase-8 (sc-5263), Proliferating cell nuclear antigen (PCNA; sc-56), androgen receptor (AR; sc-816), and β-actin (sc-81178) were purchased from Santa Cruz Biotechnology, Inc. (TX, USA). Antibody against prostate-specific antigen (PSA; PB9259) was procured from Boster Biological Technology (CA, USA). Antibodies against 5' adenosine monophosphate-activated protein kinase (AMPK; #2532) and p-AMPK (#2535) were obtained from Cell Signaling Technology (MA, USA). Total protein was extracted from prostatic tissues of rats and western blot analysis was performed as described earlier [51].

### Cell culture and sample treatment

The epithelial cell line RWPE-1 and stromal cell line WPMY-1, derived from normal human prostate tissue, were purchased from the American Type Culture Collection (ATCC; VA, USA). Cell culture for complete growth of RWPE-1 cells (lines < 20) and WPMY-1 cells (lines < 20) was performed as described earlier [52]. All cells were cultured in the charcoal-stripped fetal bovine serum or phenol red-free media to block the effects of autocrine androgens. To mimic the BPH rat model induced by androgen, seeded cells were treated with 10 nM DHT for 24 to 72 h, with or without various concentrations of BC (25–50 μM).

### MTS assay

Cell viability was determined using the CellTiter 96® AQueous One Solution Cell Proliferation Assay solution (MTS; Promega, WI, USA). The assay is based on the reduction of MTS tetrazolium to a colored formazan dye by metabolically active cells. RWPE-1 and WPMY-1 cells were seeded into a 96-well plate and treated with 3.13 to 200 μM concentration of BC for 24 h. After treatment, BC-treated cells were added along with MTS solution for 4 h, followed by measurement of cell viability by recording the absorbance of formazan dye at 490 nm using a microplate reader (Biotek; VT, USA).

### CCK-8 assay

Cell proliferation was measured utilizing the Cell Counting Kit-8 (Dojindo Molecular Technologies, Inc, D.C, USA). RWPE-1 and WPMY-1 cells were seeded into 96-well plates (1 x 10^5^ cells/well) and incubated for 24 h. The following day, the cells were starved for 24 h prior to treatment. Then, the cells were treated with 10 nM DHT, with or without various concentrations of BC (6.25–200 μM) for 24h. After treatment, CCK-8 solution was added to each well for 4 h. The number of viable cells was monitored by measuring the absorbance at a wavelength of 450 nm using an Epoch microplate reader (Biotek, Winooski, VT, USA).

### Statistical analysis

Experiments were performed in triplicate, and data are expressed as mean ± standard deviation (SD). Statistically significant values were determined using analysis of variance (ANOVA) and Dunnett's post hoc test. P-values < 0.05 are considered statistically significant. Statistical analysis was performed using the GraphPad Prism 5.
